# Lessons Learned Implementing a Protein Crystallography CURE

**DOI:** 10.1063/4.0001078

**Published:** 2025-10-27

**Authors:** Krystle J McLaughlin

**Affiliations:** Vassar College, Poughkeepsie, NY, USA

## Abstract

Course-based Undergraduate Research Experiences (CUREs) are a pedagogical approach that integrates research into the curriculum. Instead of students working through traditional teaching lab experiments with known outcomes, in a CURE students investigate an authentic research question while still learning essential lab skills. CUREs are an effective route for exposing undergraduate students to authentic research experiences, which are known to have an overall positive effect on educational outcomes. Here I will discuss challenges and successes through the development and implementation of a biochemistry CURE centered on protein research. Additionally I will explore strategies for integrating manuscript writing into a CURE, as this protein crystallography CURE was run for three semesters and has resulted in three publications with undergraduates as primary authors.

